# Comparing Coronary Artery Calcium Scoring With Cardiac Risk Scores for Predicting Cardiovascular Events in Primary Care Patients in Saudi Arabia

**DOI:** 10.7759/cureus.50120

**Published:** 2023-12-07

**Authors:** Amani A Alsulami, Abdullah H Alkhenizan, Kossay Elabd, Yaser Alendijani, Nora Alalem

**Affiliations:** 1 Family Medicine, King Faisal Specialist Hospital and Research Centre, Riyadh, SAU; 2 Family Medicine and Polyclinics, King Faisal Specialist Hospital and Research Centre, Riyadh, SAU

**Keywords:** coronary artery calcium scoring, primary healthcare, cardiovascular diseases, triglyceride glucose index, arteriosclerotic cardiovascular disease

## Abstract

Background

Cardiovascular diseases are the leading cause of death in Saudi Arabia, and cardiac risk-stratification scoring methods are critical in the primary healthcare setting to predict and potentially prevent the fatal outcomes of CVD. Therefore, this study aimed to examine the prognostic value of coronary artery calcium scoring (CACS) and other cardiac risk-stratification scores: arteriosclerotic cardiovascular disease (ASCVD) risk estimator, cardiovascular risk score (QRISK2), and triglyceride glucose index (TyG) in primary healthcare facilities in Riyadh, Saudi Arabia.

Methods

A retrospective cohort study was conducted at Family Medicine Clinics, and data on patient’s demographics, medical records, and chronic illnesses obtained from the Integrated Clinical Information System (ICIS) database that were recorded between 2010 and 2019 were analyzed. We performed descriptive statistics, student's t-test, analysis of variance (ANOVA), Pearson correlation, Cohen's Kappa, and regression analyses.

Results

QRISK (p<0.001) and ASCVD (p<0.05) risk estimators positively correlated with the CACS score in predicting fatal and non-fatal cardiac outcomes while the TyG score had the lowest prediction ability among all the other risk estimators. CACS (OR = 1.003; 95% CI: 1.005 -1.002) (p<0.001), ASCVD (OR = 18.177; 95%CI: 214.578 - 1.540) (p=0.021), and QRISK2 (OR=154.796; 95%CI: 4137.356 - 5.792) (p=0.003) significantly predict stenosis unlike the TyG score's statistically insignificant prediction (p>0.05).

Conclusion

These findings show that ASCVD and QRISK2 are consistent with CACS and are effective risk indicators that could be used to predict cardiac-associated fatal and non-fatal cardiac events among primary care patients. This indicates that the integration of multiple risk scores, as necessary, can all contribute to more effective risk assessment and prevention of coronary artery diseases and related cardiovascular events.

## Introduction

Cardiovascular diseases (CVDs) are the leading cause of mortality globally [1]. In Gulf Cooperation Council (GCC) countries, especially in the Kingdom of Saudi Arabia (KSA), coronary heart disease (CHD) is the leading cause of death, with a prevalence previously reported to be 5.5% [2-4]. A previous report indicated that the prevalence of CHD in Saudi Arabia is 5.5%. CVDs are reported to account for over 45% of the mortality rate in Saudi Arabia [5]. Coronary artery calcification (CAC), which is the buildup of calcium in the arteries [6], is highly prevalent in patients with CHD and is associated with significant adverse cardiovascular events and the development of advanced atherosclerosis [4,7]. The prevalence of CAC in Saudi Arabia is high in patients with normal myocardial perfusion imaging (MPI) and is associated with risk factors such as age, male sex, and diabetes [8]. CHD risk stratification methods, such as atherosclerotic cardiovascular disease (ASCVD) score, cardiovascular risk score (QRISK2), and triglycerides glucose (TyG) index [9,10] are used to predict CHD and CVD risks. However, while it is still a relatively new method compared to other risk estimators, the coronary artery calcium score (CACS) is considered the gold-standard method to assess CAC [4,9,11-14]. Additionally, CACS is considered an independent indicator of cardiovascular events and superior to the other available risk estimators [11,15,16]. The Framingham Risk Score (FRS) predicts the risk of coronary disease based on age, gender, smoking history, blood pressure, cholesterol, high-density lipoprotein cholesterol (HDL-C), and blood glucose levels or diabetes history [17,18]. Combined with the FRS, it was found that high CACS can alter predicted risk based on FRS alone, particularly in patients with intermediate risk, where clinical decision-making is most uncertain [17]. It has been reported that a combined CAC and ASCVD risk assessment can guide systolic blood pressure control measures, particularly among individuals with an ASCVD risk of 5-15% and pre-hypertension or mild hypertension [19].

It was previously reported that asymptomatic Saudis, especially women, who underwent CACS screening had a higher chance of developing a heart attack than the rest of the world population [20,21], which highlights the vital need to utilize the currently available and non-invasive screening methods to predict and prevent fatal and non-fatal cardiac events in Saudi Arabia. Therefore, this study examined CACS and other cardiac risk stratification scores, comparing their prediction of fatal and non-fatal cardiac events among primary care patients in King Faisal Specialist Hospital (KFSH), Family Medicine Department, in Riyadh, Saudi Arabia.

## Materials and methods

Study design and data source

We retrospectively retrieved 2010-2019 electronic health records from the Integrated Clinical Information System (ICIS) database of all adult patients (n=404) who were regularly seen at Family Medicine & Polyclinics clinics at King Faisal Specialist Hospital and Research Centre (KFSH&RC) in Riyadh. ICIS is a comprehensive digital patient-care system that automates and connects all patient-related information. ICIS possesses a local electronic client care record that contains comprehensive operational and strategic information. This record has the capability to access information from other important information systems through various system interfaces that are linked to specific client identifiers.

Participants

This retrospective cohort study included asymptomatic patients without known coronary heart disease who had been referred for CAC screening because of the presence of one or more coronary artery disease (CAD) risk factors (diabetes, hypertension (HTN), hypercholesterolemia, family history of CAD, and obesity). We excluded patients below the age of 18, patients with myocardial infarction or coronary revascularization, patients on dialysis, patients with elevated triglyceride levels (≥400 mg/dL), patients taking cholesterol-lowering medications, patients with known peripheral vascular disease, heart failure, and familial hypercholesterolemia.

Asymptomatic patients were defined as individuals who did not exhibit any clinical manifestations, such as chest pain, dyspnea, or other associated symptoms and did not have a documented history or previous diagnosis of CVD, indicating that their condition remains undetectable through standard clinical assessments.

We recorded all data on demographic factors, such as age, gender, nationality, weight, height, body mass index (BMI), and smoking history from medical records. The age was calculated in years, gender was binary (male and female), weight was calculated in kilograms (kg), height in meters (m), and BMI was calculated as a ratio of weight over height squared (kg/m^2^).

Patients’ medical history of chronic illness

We recorded data on the medical history of patients with chronic illnesses known to be risk factors for CAD, and medication history. High blood pressure (HTN), which was defined by either self-reporting HTN, currently taking anti-hypertensive drugs, or recorded systolic blood pressure (SBP) ≥140 mmHg, and/or diastolic blood pressure (DBP) ≥90 mmHg for three or more consecutive times or based on ambulatory BP (24hr BP) [22].

We recorded patients' history of CAD prior to and after CACS as well as fatal and non-fatal myocardial infarction, family history of ischemic heart disease, premature cardiac death in a first-degree relative (women less than 65 and men less than 55), chronic kidney disease (CKD) and stage assessed based on estimated glomerular filtration rate (eGFR) (CKD stages: 1. Normal: >90, 2. Mild CKD: 60-89 ml/min, 3A. Moderate CKD: 45-59 ml/min, 3B. Moderate CKD: 30-44 ml/min, 4. Severe CKD: 15-29 ml/min, end-stage CKD, 5: <15 ml/min) [23], rheumatoid arthritis, atrial fibrillation, primary vessel affected identified by CACS, and degree of occlusion. In addition, obesity was assessed and defined as BMI > 30. Abnormal lipids (higher triglycerides, low-density lipoprotein, lower high-density lipoprotein), and diabetes mellitus type 2 were measured either as fasting serum glucose ≥7.0 mmol/L, two-hour serum glucose using oral glucose tolerance test ≥11.1 mmol/L, or HbA1c ≥6.5 currently using hypoglycemic drugs or are taking daily insulin injections.

Risk score measurements

At the time of visits, all patients' blood samples were taken after 8-12 hours of fasting. Data of fasting plasma glucose (FPG), hemoglobin A1C, total cholesterol (TC), high-density lipoprotein cholesterol (HDL-C), triglyceride (TG), and low-density lipoprotein cholesterol (LDL-C) that had been collected at the time of the visits for CACS were retrieved from laboratory records. The TyG index was calculated as follows: [fasting triglycerides (mg/dL) × fasting plasma glucose (mg/dL)/2] [24], and the TyG index cutoff was 4.49. ASCVD and QRISK2 scores were calculated using commercially available calculators on their websites. 

Computed tomography (CT) calcium scoring measurement

The extent of calcification was quantified using the Agatston score, which was calculated by multiplying the area of calcification by the corresponding density [25]. An Agatston score of >0 reported CAC presence. A CACS between 0 and 100 (mild disease), 101 and 400 (moderate disease), and more than 400 (severe disease) [4,6,7,10,26].

Statistical analysis

SAS software, version 9.4 (Statistical Analysis System, SAS Institute Inc., Cary, NC, USA), was used to perform all the statistical analyses reported in this study. Descriptive statistics were summarized as frequencies and percentages. The primary outcome variables, including CAC scoring, ASCVD, QRISK2, and TyG, were comparatively analyzed using the chi-square and Fisher-Freeman-Halton exact tests. Pearson correlation was used to measure the strength of the linear relationship between the CAC score and the other cardiac risk stratification scores while Cohen's Kappa was used to assess the level of agreement. Logistic regression was used to assess the effect of risk factors on the possibility of developing cardiac events. The level of statistical significance was set at p<0.05.

Ethical considerations

This study is non-interventional, and it did not cause any harm to the patients. Ethical approval was obtained from the Office of Research Affairs (ORA) at KFSH&RC before the initiation of the study (RAC# 2211013), and a waiver of consent was granted.

## Results

Patients' demographics and medical history 

Of all records obtained between January 2010 and December 2019, only 404 patients were eligible and included in this study. As shown in Table 1, the included patients were predominantly male (n=262; 64.9%). About half of the responders (n=192; 47.5%) suffered from cardiac symptoms, with chest pain being the most common symptom (n=123; 30.4%). Most patients (n=165; 65.6%) had a desirable amount of cholesterol while only 44 (10.9%) had high cholesterol levels. Surprisingly, there was about equal distribution of patients with (n=206; 51%) and without (n=198; 49%) HTN, as well as with (n=175; 43.3%) and without (n=227; 56.2%) clinical obesity. Nevertheless, most patients had DLD (n=274; 67.8%).

**Table 1:** Overall patients’ demographics and medical health history

Variables	Frequency (%)
Gender	
Male	262(64.9)
Female	142(35.1)
Nationality	
Saudi	256(63.4)
Non-Saudi	148(36.6)
Smoking history	
Yes	79(19.6)
No	312(77.2)
Undocumented	13(3.2)
Cardiac symptoms history	
Yes	192(47.5)
No	210(52.0)
Not documented	2(0.5)
Symptoms category (194(48.0))	
Chest pain	123(30.4)
Dyspnea	32(7.9)
Palpitation	9(2.2)
Other	6(1.5)
Combination of the above	24(5.9)
Total cholesterol	
Desirable	265(65.6)
Borderline high	95(23.5)
High	44(10.9)
Low-density lipoprotein cholesterol (LDL-C)	
Optimal	100(24.8)
Near-optimal	128(31.7)
Borderline	88(21.8)
High	72(17.8)
Very high	16(4.0)
Hypertension history	
Yes	206(51.0)
No	198(49.0)
Obesity (BMI>30)	
Yes	175(43.3)
No	227(56.2)
Dyslipidemia	
Yes	274(67.8)
No	130(32.2)
Diabetes mellitus type 2	
Yes	155(38.4)
No	249(61.6)
Chronic kidney disease	
Yes	12(3.0)
No	392(97.0)
Coronary revascularization or stroke	
Yes	40(9.9)
No	363(89.9)
History of atrial fibrillation	
Yes	7(1.7)
No	397(98.3)
History of rheumatoid arthritis	
Yes	4(1)
No	400(99.0)
Family history of ischemic heart disease	
Yes	83(20.5)
No	208(51.5)
Family history of premature cardiac death in a first-degree relative	
Yes	25(6.2)
No	239(59.2)

Demographic and baseline characteristics among different CAC scores

This study evaluated the relationship between CAC scores and the participants' demographics and medical history. As shown in Table 2, the CAC scoring system compares mild, moderate, and severe risks to predict the patients' cardiac outcomes and guide the intensity of their medical management. There were significant differences (p<0.05) between risk levels (shown by CACS gradings) and levels of LDL, HTN, and DLD, previous history of heart disease, arterial fibrillation, cardiac disease history, stenosis, and CAD. However, the CAC scoring system could not detect any significant difference in total cholesterol levels, obesity, or diabetes mellitus (Type 2) among the three groups based on demographics.

**Table 2:** Relationship between baseline characteristics of the study population and CACS *Statistically significant, p<0.05 The data have been represented as %. CACS: coronary artery calcium score

Variables	CACS						P-value	
	Mild		Moderate			Severe		
Gender								
Male	85.9%		8.8%			5.3%	0.277	
Female	85.9%		5.6%			8.5%		
Nationality								
Saudi	83.6%		9.8%			6.6%	0.107	
Non-Saudi	89.9%		4.1%			6.1%		
Smoking								
Yes	84.8%		10.1%			5.1%	0.642	
No	85.9%		7.4%			6.7%		
Cardiac symptoms								
Yes	82.8%		8.3%			8.9%	0.131	
No	89.0%		6.7%			4.3%		
Total cholesterol grade								
Desirable	83.0%		9.4%			7.5%	0.183	
Borderline high	89.5%		5.3%			5.3%		
High	95.5%		2.3%			2.3%		
LDL grade								
Optimal	76.0%		14.0%			10.0%	0.012*	
Near-optimal	82.8%		8.6%			8.6%		
Borderline	92.0%		3.4%			4.5%		
Presence of hypertension								
Yes	76.7%		14.1%			9.2%	0.000*	
No	95.5%		1.0%			3.5%		
Presence of obesity								
Yes	82.3%		9.1%			8.6%	0.183	
No	88.5%		6.6%			4.8%		
Presence of dyslipidemia								
Yes	81.4%		10.2%			8.4%	0.001*	
No	95.4%		2.3%			2.3%		
Presence of diabetes mellitus								
Yes	81.3%		10.3%			8.4%	0.110	
No	88.8%		6.0%			5.2%		
Presence of chronic kidney disease (CKD)								
Yes	75.0%		8.3%			16.7%	0.335	
No	86.2%		7.7%			6.1%		
If CKD is present, was the patient on dialysis								
Yes	100.0%		0.0%			0.0%	0.982	
No	96.6%		1.7%			1.7%		
History of previous heart diseases								
Yes		62.5%	20.0%		17.5%			0.000*
No		88.7%	6.1%		5.2%			
Presence of arterial fibrillation								
Yes		42.9%	28.6%		28.6%			0.004*
No		86.6%	7.3%		6.0%			
Presence of rheumatoid arthritis								
Yes		100.0%	0.0%		0.0%			0.718
No		85.8%	7.8%		6.5%			
Family history of cardiac disease								
Yes		85.5%	4.8%		9.6%			0.026*
No		92.3%	5.3%		2.4%			
Presence of coronary stenosis after the CAC study								
Yes	15.0%		60.9%	0.0%				0.000*
No	85.0%		39.1%	0.0%				
If the patient had CAD after the CAC study								
Yes	3.8%		26.7%	53.8%				0.000*
No	96.2%		73.3%	46.2%				
If the patient had a stroke after the CAC study								
Yes	0.6%		0.0%	0.0%				0.847
No	99.4%		100.0%	100.0%				

Next, the study assessed the relationship between ASCVD scores and the participants' demographics and medical history. Baseline demographic and clinical data variables of the study participants were stratified into groups according to ASCVD scores, with four scales (low, borderline, intermediate, and high) (Table 3). There was a significant relationship between ASCVD scores and grades of LDL, HTN, DLD, diabetes mellitus (Type 2), family history of cardiac disease, stenosis, and CAD (p<0.05).

**Table 3:** Relationship between baseline characteristics of the study population and ASCVD scores * Statistically significant, p<0.05 The data have been represented as %. ASCVD: arteriosclerotic cardiovascular disease; CACS: coronary artery calcium score

Variables	ASCVD						P-value
	Low	Borderline		Intermediate	High		
Gender							
Male	22.4%		14.7%	41.7%		21.2%	0.000*
Female	53.5%		9.9%	30.3%		6.3%	
Nationality							
Saudi	27.6%		13.4%	40.9%		18.1%	0.011*
Non-Saudi	43.5%		12.2%	32.0%		12.2%	
Smoking							
Yes	19.0%		11.4%	43.0%		26.6%	0.002*
No	37.5%		13.3%	35.9%		13.3%	
Cardiac symptoms							
Yes	30.7%		11.6%	37.6%		20.1%	0.173
No	35.7%		14.3%	37.6%		12.4%	
Total cholesterol grade							
Desirable	30.5%		13.0%	40.1%		16.4%	0.252
Borderline high	36.8%		13.7%	37.9%		11.6%	
High	43.2%		11.4%	22.7%		22.7%	
LDL grade							
Optimal	18.6%		18.6%	45.4%		17.5%	0.022*
Near-optimal	37.5%		11.7%	32.0%		18.8%	
Borderline	36.4%		10.2%	43.2%		10.2%	
High	43.1%		9.7%	34.7%		12.5%	
Presence of hypertension							
Yes	21.1%		9.8%	44.6%		24.5%	0.000*
No	46.2%		16.2%	30.5%		7.1%	
Presence of obesity							
Yes	29.5%		13.3%	41.0%		16.2%	0.504
No	36.3%		12.8%	35.0%		15.9%	
Presence of dyslipidemia							
Yes	25.4%		14.3%	41.9%		18.4%	0.000*
No	50.4%		10.1%	28.7%		10.9%	
Diabetes mellitus							
Yes	16.3%		11.1%	41.8%		30.7%	0.000*
No	44.0%		14.1%	35.1%		6.9%	
Chronic kidney disease							
Yes	16.7%		16.7%	50.0%		16.7%	0.643
No	33.9%		12.9%	37.3%		15.9%	
Dialysis							
Yes	0.0%		100.0%	0.0%		0.0%	0.139
No	47.0%		14.8%	31.3%		7.0%	
History of heart disease							
Yes	17.9%		15.4%	38.5%		28.2%	0.056
No	35.2%		12.7%	37.7%		14.4%	
Arterial fibrillation							
Yes	0.0%		28.6%	28.6%		42.9%	0.069
No	34.0%		12.7%	37.8%		15.5%	
Rheumatoid arthritis							
Yes	50.0%		0.0%	50.0%		0.0%	0.645
No	33.2%		13.1%	37.5%		16.1%	
Family history of cardiac disease							
Yes	45.8%		16.9%	21.7%		15.7%	0.021*
No	31.1%		13.6%	39.8%		15.5%	
Stenosis							
Yes	14.3%		30.0%	21.3%		48.6%	0.001*
No	85.7%		70.0%	78.7%		51.4%	
if the patient has CAD after CACS							
Yes	4.5%		5.8%	8.7%		19.0%	0.006*
No	95.5%		94.2%	91.3%		81.0%	
if the patient has a stroke after CACS							
Yes	0.7%		0.0%	0.0%		1.6%	0.452
No	99.3%		100.0%	100.0%		98.4%	

In addition, the relationship between QRISK2 and patients' demographics and medical history is shown in Table 4. Like the CAC scoring system, the QRISK2 estimator uses a low, moderate, and high scaling system. We found a statistically significant relationship between QRISK2 scores and cardiac symptoms, total cholesterol grade, HTN, DLD, diabetes mellitus (Type 2), previous history of heart disease, arterial fibrillation, stenosis, and CAD.

**Table 4:** Relationship between baseline characteristics of the study population and QRISK2 *Statistically significant, p<0.05 The data have been represented as %. QRISK2: cardiovascular risk score; CACS: coronary artery calcium score

Variables	QRISK2			P-value
	Low	Moderate	High	
Gender				
Male	52.9%	30.7%	16.5%	0.001*
Female	66.2%	29.6%	4.2%	
Nationality				
Saudi	48.2%	36.5%	15.3%	0.000*
Non-Saudi	73.6%	19.6%	6.8%	
Smoking				
Yes	44.9%	33.3%	21.8%	0.005*
No	61.2%	28.8%	9.9%	
Cardiac symptoms				
Yes	51.0%	32.8%	16.1%	0.019*
No	63.2%	28.2%	8.6%	
Total cholesterol grade				
Desirable	54.5%	32.2%	13.3%	0.329
Borderline high	66.3%	23.2%	10.5%	
High	56.8%	34.1%	9.1%	
LDL grade				
Optimal	49.0%	34.0%	17.0%	0.150
Near-optimal	55.1%	32.3%	12.6%	
Borderline	64.8%	30.7%	4.5%	
High	65.3%	20.8%	13.9%	
Very high	56.2%	31.2%	12.5%	
Prescence of hypertension				
Yes	42.7%	38.8%	18.4%	0.000*
No	73.1%	21.3%	5.6%	
Presence of obesity				
Yes	50.9%	34.9%	14.3%	0.068
No	62.4%	27.0%	10.6%	
Presence of dyslipidemia				
Yes	50.0%	36.1%	13.9%	0.000*
No	73.6%	17.8%	8.5%	
Presence of diabetes mellitus				
Yes	36.1%	39.4%	24.5%	0.000*
No	71.0%	24.6%	4.4%	
Presence of chronic kidney disease				
Yes	33.3%	50.0%	16.7%	0.217
No	58.3%	29.7%	12.0%	
History of dialysis				
Yes	0.0%	100.0%	0.0%	0.051
No	81.9%	13.8%	4.3%	
History of heart diseases				
Yes	40.0%	32.5%	27.5%	0.003*
No	59.7%	30.1%	10.2%	
History of arterial fibrillation				
Yes	42.9%	14.3%	42.9%	0.041*
No	57.8%	30.6%	11.6%	
History of rheumatoid arthritis				
Yes	100.0%	0.0%	0.0%	0.328
No	57.2%	30.5%	12.2%	
Family history of cardiac disease				
Yes	70.7%	20.7%	8.5%	0.285
No	61.1%	26.0%	13.0%	
Stenosis				
Yes	18.7%	22.8%	51.6%	0.001*
No	81.3%	77.2%	48.4%	
If the patient had CAD after the CAC study				
Yes	5.2%	10.7%	20.8%	0.001*
No	94.8%	89.3%	79.2%	
If the patient had a stroke after the CAC study				
Yes	0.4%	0.0%	2.0%	0.225
No	99.6%	100.0%	98.0%	

Finally, we assessed the relationship between the TyG scoring system and the patient's demographics and medical history. As shown in Table 5, this risk estimator has only a low and high grading scale. We found a significant relationship (p<0.05) between TyG scales, DLD levels, and type 2 diabetes mellitus.

**Table 5:** Relationship between the baseline characteristics of the study population and TyG * Statistically significant, p<0.05 The data have been represented as %. TyG: triglyceride glucose index; CAC: coronary artery calcium

Variables	TyG		P-value
	Low	High	
Gender			
Male	22.4%	14.7%	0.157
Female	53.5%	9.9%	
Nationality			
Saudi	25.0%	75.0%	0.113
Non-Saudi	18.4%	81.6%	
Smoking			
Yes	22.8%	77.2%	0.658
No	20.5%	79.5%	
Cardiac symptoms			
Yes	18.8%	81.2%	0.369
No	22.4%	77.6%	
Total cholesterol grade			
Desirable	20.0%	80.0%	0.388
Borderline high	25.3%	74.7%	
High	15.9%	84.1%	
LDL grade			
Optimal	29.0%	71.0%	0.158
Near-optimal	17.2%	82.8%	
Borderline	17.0%	83.0%	
High	22.2%	77.8%	
Presence of hypertension			
Yes	17.5%	82.5%	0.094
No	24.2%	75.8%	
Presence of obesity			
Yes	17.7%	82.3%	0.202
No	22.9%	77.1%	
Presence of dyslipidemia			
Yes	16.8%	83.2%	0.004*
No	29.2%	70.8%	
Presence of diabetes mellitus			
Yes	13.5%	86.5%	0.005*
No	25.3%	74.7%	
Presence of chronic kidney disease			
Yes	8.3%	91.7%	0.280
No	21.2%	78.8%	
Dialysis			
Yes	0.0%	100.0%	0.549
No	26.5%	73.5%	
Previous history of heart diseases			
Yes	25.0%	75.0%	0.495
No	20.4%	79.6%	
Arterial fibrillation			
Yes	28.6%	71.4%	0.609
No	20.7%	79.3%	
Rheumatoid arthritis			
Yes	50.0%	50.0%	0.148
No	20.5%	79.5%	
Family history of cardiac disease			
Yes	18.1%	81.9%	0.131
No	26.4%	73.6%	
Stenosis			
Yes	25.0%	24.3%	0.927
No	75.0%	75.7%	
If the patient had CAD after the CAC study			
Yes	7.1%	9.1%	0.568
No	92.9%	90.9%	
If the patient had a stroke after the CAC study			
Yes	1.2%	0.3%	0.309
No	98.8%	99.7%	

Level of agreement and correlation between CACS and the other cardiac risk scores

Cohen's Kappa coefficient revealed an almost perfect level of concordance between CACS and QRISK2, suggesting strong consistency between the two risk stratification tools. Additionally, a moderate level of agreement was observed between CACS and ASCVD, indicating a reasonable degree of alignment between these tools. Table 6 shows that QRISK2 (p<0.001) and ASCVD (P=0.003) significantly correlated with the CAC scores, which indicates that QRISK2 and ASCVD are consistent with CACS in risk prediction. There was no significant correlation detected between the CAC score and the TyG index. In addition, the CAC score had a significant level of agreement with both the ASCVD (P=0.011) and QRISK2 score (p=0.008), but not with the TyG index (Table 7).

**Table 6:** Correlations between CACS and risk estimators * Statistically significant, p<0.05 CACS: coronary artery calcium score

Variables					
			TyG index N=404	QRISK2 N=401	ASCVD N=397
CACS	r	0.074		0.247	0.147
	P-value	0.136		<0.001*	0.003*

**Table 7:** Level of agreement between CACS and risk estimators *Statistically significant, p<0.05 CACS: coronary artery calcium score

Variables compared to CACS	Kappa	P-value
TyG	-0.002	0.880
QRISK2	0.080	0.008*
ASCVD	0.049	0.011*

Cardiac risk scores in predicting stenosis

The ability of cardiac risk estimators to predict stenosis was assessed using univariate logistic regression analysis (Table 8) and found that CACS (OR= 1.003; 95% CI: 1.005 -1.002) (p<0.001), ASCVD (OR= 18.177; 95%CI: 214.578 -1.540) (p=0.021), and QRISK2 (OR= 154.796; 95%CI: 4137.356 - 5.792) (p=0.003) were able to predict stenosis. However, based on the analysis, the TyG Index did not independently predict stenosis (p=0.231).

**Table 8:** Univariate analysis evaluating the association between stenosis and risk estimators * Statistically significant, p<0.05 TyG: triglyceride glucose index; QRISK2: cardiovascular risk score; ASCVD: arteriosclerotic cardiovascular disease; CACS: coronary artery calcium score

Risk Estimators	Univariate	
	OR (95% CI)	P-value
TyG index	1.853 (5.085 - 0.675)	0.231
QRISK2	154.796 (4137.356 - 5.792)	0.003*
ASCVD	18.177 (214.578 - 1.540)	0.021*
CACS	1.003 (1.005 -1.002)	0.000*

## Discussion

This retrospective cohort study evaluated the prognostic gold-standard CAC scoring method known as CACS and compared it with other risk estimators in primary health care in Riyadh, Saudi Arabia. Although the CACS score is considered the gold-standard method to detect CAC events in patients [4,9,11-14], it is still unknown whether the combination of CACS and other risk scores can improve fatal and non-fatal cardiac events associated with CAC. To the best of our knowledge, this is the first study to compare CACS with other risk scores, such as ASCVD, QRISK2, and the TyG index, in their ability to predict CAC events.

Several studies have evaluated each score individually or in combination with other risk estimators. Pereira et al. demonstrated that when the CAC score was combined with other scores, such as QRISK2 and FRS, they improved the risk assessment in HIV patients [27]. Likewise, others have demonstrated that using the ASCVD score can improve CAC prediction and thus enhance the type of treatment provided to CAC patients [28]. Kapelios et al. reported that CACS was a highly effective indicator for identifying various levels of subclinical CAD in asymptomatic people living with HIV without CVD [29]. CACS proved to be a highly effective indicator for identifying various levels of subclinical CAD. Given these observations, it is evident that using multiple risk scores and estimators enhances the detection and prediction of fatal and non-fatal cardiovascular events in CAC patients [9,21,27].

The current study evaluated the relationship between the baseline characteristics and CACS, ASCVD, QRISK2, and TyG index. We found that the CACS scores were significantly associated with LDL, HTN, DLD, heart disease history, arterial fibrillation, cardiac disease history, stenosis, and CAD. This means that CACS could predict the risks of these conditions. However, CACS was not associated with diabetes mellitus, which was otherwise associated with the ASCVD score. There was no relationship between the ASCVD score and heart disease history or arterial fibrillation. Nevertheless, the TyG index could only detect a significant difference in DLD levels and type 2 diabetes mellitus. These results indicate that the TyG index is the least informative in predicting cardiac outcomes out of the four scores assessed in this study. Previous studies have indicated that the TyG index predictive value varies across populations (highly predictive among morbidly obese individuals than other categories [30,31]. Our findings showed that the CAC score positively correlated and highly agreed with the ASCVD score and QRISK2 score but not with the TyG index. This observation could be explained by the fact that the TyG index predominantly detects insulin resistance in patients [32]. However, some studies argue that the TyG index could be used to predict cardiovascular events [9].

Identifying severe occult coronary artery stenosis helps improve the prevention of cardiac events [33]. Thus, the current study demonstrated that stenosis could be predicted using CACS, ASCVD, and QRISK2 but not the TyG index. This suggests that TyG is the least informative stratification strategy when predicting stenosis in CAC patients. Additionally, this study showed that CACS, ASCVD, and QRISK2 risk estimators complement each other to predict CAC events in patients in primary healthcare settings. These observations are consistent with previous studies, which reported that using the CACS score improves stenosis prediction in patients and thus can be used to prevent the progression to developing severe cardiac events such as heart attack [29,33]. This is an additional study comparing CACS scores with other risk scores (ASCVD, QRISK) and their ability to prevent cardiovascular events. The finding that the TyG index was the least predictive of all was also reported by a previous study [34].

The findings highlight that the integration of multiple risk scores, careful consideration of baseline characteristics, and recognition of specific indices' limitations can all contribute to more effective risk assessment and prevention strategies in the context of coronary artery calcification and related cardiovascular events. This study used a clinical and local database, which is vital to local data use and enhancing locally contextualized evidence from understudied populations.

This study was limited to a single healthcare facility in Riyadh, Saudi Arabia, with a small sample size relative to Riyadh’s population. Additionally, it was a retrospective study, which is prone to selection bias and missing or inaccurate information. Thus, further extensive longitudinal studies with a larger and more diverse population are recommended. Moreover, future research should look into applying our findings in different healthcare settings and populations to strengthen the evidence base for risk assessment and prevention strategies.

## Conclusions

This study identified that combined with the gold-standard CAC score, the ASCVD and QRISK scores are valid indicators of fatal and non-fatal cardiac events among primary care patients. QRISK2 and ASCVD are consistent with CACS in predicting the risks. ASCVD and QRISK2 (but not TyG) can also assess and detect stenosis. These findings indicate that a combination of CACS and other risk scores can improve fatal and non-fatal cardiac events. This study could guide primary healthcare providers in a better choice of risk estimation methods to assess coronary artery plaque and predict their patients’ cardiac outcomes to guide medical management and improve the quality of care. Future studies could use the findings of this study as a baseline and further explore the different risk indicators among demographically different populations to further inform measures designed to optimize health outcomes among CVD patients.
